# Texture‐based morphometry in relation to apolipoprotein ε4 genotype, ageing and sex in a midlife population

**DOI:** 10.1002/hbm.26798

**Published:** 2024-07-31

**Authors:** Maria‐Eleni Dounavi, Elijah Mak, Gregory Operto, Graciela Muniz‐Terrera, Katie Bridgeman, Ivan Koychev, Paresh Malhotra, Lorina Naci, Brian Lawlor, Li Su, Carles Falcon, Karen Ritchie, Craig W. Ritchie, Juan Domingo Gispert, John T. O'Brien

**Affiliations:** ^1^ Department of Psychiatry School of Clinical Medicine, University of Cambridge Cambridge UK; ^2^ Barcelonaβeta Brain Research Center (BBRC), Pasqual Maragall Foundation Barcelona Spain; ^3^ Centre for Dementia Prevention University of Edinburgh Edinburgh UK; ^4^ Heritage College of Osteopathic Medicine Ohio University Athens Ohio USA; ^5^ Department of Psychiatry Oxford University UK; ^6^ Division of Brain Science Imperial College Healthcare NHS Trust UK; ^7^ Institute of Neuroscience, Trinity College Dublin, University of Dublin Ireland; ^8^ Department of Neuroscience University of Sheffield Sheffield UK; ^9^ INSERM and University of Montpellier Montpellier France

**Keywords:** ageing, Alzheimer's disease, APOE4, dementia risk, morphometry, MRI, texture

## Abstract

Brain atrophy and cortical thinning are typically observed in people with Alzheimer's disease (AD) and, to a lesser extent, in those with mild cognitive impairment. In asymptomatic middle‐aged apolipoprotein ε4 (ΑPOE4) carriers, who are at higher risk of future AD, study reports are discordant with limited evidence of brain structural differences between carriers and non‐carriers of the ε4 allele. Alternative imaging markers with higher sensitivity at the presymptomatic stage, ideally quantified using typically acquired structural MRI scans, would thus be of great benefit for the detection of early disease, disease monitoring and subject stratification. In the present cross‐sectional study, we investigated textural properties of T1‐weighted 3T MRI scans in relation to APOE4 genotype, age and sex. We pooled together data from the PREVENT‐Dementia and ALFA studies focused on midlife healthy populations with dementia risk factors (analysable cohort: 1585 participants; mean age 56.2 ± 7.4 years). Voxel‐based and texture (examined features: contrast, entropy, energy, homogeneity) based morphometry was used to identify areas of volumetric and textural differences between APOE4 carriers and non‐carriers. Textural maps were generated and were subsequently harmonised using voxel‐wise COMBAT. For all analyses, APOE4, sex, age and years of education were used as model predictors. Interactions between APOE4 and age were further examined. There were no group differences in regional brain volume or texture based on APOE4 carriership or when age × APOE4 interactions were examined. Older people tended to have a less homogeneous textural profile in grey and white matter and a more homogeneous profile in the ventricles. A more heterogeneous textural profile was observed for females in areas such as the ventricles, frontal and parietal lobes and for males in the brainstem, cerebellum, precuneus and cingulate. Overall, we have shown the absence of volumetric and textural differences between APOE4 carriers and non‐carriers at midlife and have established associations of textural features with ageing and sex.

## Graphical abstract

In this study, we investigated textural brain changes in relation to a risk gene for sporadic late‐onset dementia (apolipoprotein ε4‐APOE4), ageing and sex at midlife. We found no differences between APOE4 carriers and non‐carriers, suggesting the absence of subtle structural alterations, and we established associations between brain texture, age and sex.

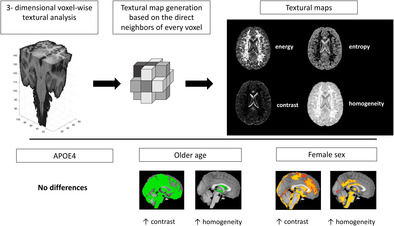

**Practitioner Points**

- Texture‐based morphometry was applied to detect areas of brain textural differences between carriers and non‐carriers of the apolipoprotein ε4 gene.
- There were no differences in the textural profile of carriers and non‐carriers.
- Older age and female/male sex were associated with distinct brain textural patterns.

## 1 INTRODUCTION

Alzheimer's disease (AD) manifests itself in the brain with distinct pathological signatures involving amyloid plaque deposition, neurofibrillary tau tangles and neurodegeneration, giving rise to the A/T/N (amyloid/tau/neurodegeneration) pathological classification framework (Jack et al., 2018). From these pathological hallmarks, neurodegeneration can be evaluated using structural MRI by measuring regional brain volumes. In the disease's trajectory, neurodegeneration as captured based on structural MRI occurs during the prodromal disease stage—mild cognitive impairment (MCI) and is typically localised in the medial temporal lobe, though atypical presentations also exist (Cedres et al., 2020; Ferreira et al., 2017). Structural MRI scans lend themselves to analysis beyond regional volumetry, allowing for the quantification of measures which could be sensitive to disease pathology earlier compared to commonly quantified structural measures such as brain volume. Such features quantified from structural scans involve but are not limited to textural features (Kassner & Thornhill, 2010), grey matter (GM)/morphometric networks (Dicks et al., 2020; Pichet Binette et al., 2020), fractal dimension (Ziukelis et al., 2022) and measures capturing cortical gyrification patterns (Núñez et al., 2020).

Amongst them, textural features have been shown to yield high classification accuracy in tasks seeking to determine participants with AD or a higher likelihood of progressing to dementia from MCI and to confer an additive predictive benefit to volumetry (Lee et al., 2019; Luk et al., 2018; Sørensen et al., 2016, 2017). In addition, it has been shown that future amyloid accumulation in MCI could be predicted by models incorporating radiomics/textural features (Kim et al., 2021). Hence, it can be hypothesised that texture could be more sensitive in detecting subtle changes in the brain compared to more traditional volumetric approaches. Texture can be evaluated using multiple different methods, some of which have a direct conceptual translatability (e.g., image contrast), whereas others rely on complex mathematical models which are not necessarily easily perceivable (e.g., Gabor features) (Cai et al., 2020; Haralick et al., 1973; Zacharaki et al., 2009). Amongst the most popular textural features in medical image analysis are the Haralick features, measuring properties such as image contrast and homogeneity (Haralick et al., 1973). These properties are quantified by establishing the pattern of spatial co‐occurrence of individual intensities using a statistical framework. Haralick features, or else the grey level co‐occurrence matrix (GLCM) technique, has been used extensively in medical research (Cai et al., 2020; Kassner & Thornhill, 2010).

Textural features are typically measured within pre‐specified regions of interest (ROI) following image pre‐processing to standardise intensity values. One proposed method extended this ROI‐based framework to a voxel‐wise approach whereby instead of focusing on specific ROIs, the ROI is a sliding window, thus allowing for the generation of textural maps (Maani et al., 2015) focused on the central voxel of the ROI. Voxel‐wise analysis of texture (texture‐based morphometry—TBM) was demonstrated to be more sensitive compared to volumetry in predicting conversion from MCI to AD (Luk et al., 2018). Furthermore, it has been shown that textural properties are associated with regional tau but not amyloid burden (Lee et al., 2021). Multi‐site textural analysis studies or studies of textural alterations in relation to dementia risk factors at midlife have not been conducted to date. One such risk factor is the apolipoprotein ε4 (*APOE4*) gene, which is the main genetic risk factor for sporadic late‐onset AD. Carrying one copy of the gene confers a threefold to fourfold and two copies an 8‐ to 12‐fold risk for developing AD (Farrer et al., 1997; Neu et al., 2017).

Our goal in the present study was to evaluate whether TBM would reveal early alterations in a cognitively asymptomatic midlife cohort enriched with APOE4 carriers from two large cohort studies, the PREVENT‐Dementia programme (UK‐Ireland) (Ritchie et al., 2013; Ritchie & Ritchie, 2012) and the ALFA (ALzheimer and FAmilies) (Molinuevo et al., 2016) study (Spain). In the PREVENT cohort, we have shown that there are no prominent structural changes at baseline in APOE4 carriers (Dounavi et al., 2022). Similarly, for the first wave of scans from the ALFA cohort, there were only small regions of macrostructural differences between APOE4 carriers and non‐carriers, with areas of both atrophy and hypertrophy detected (Cacciaglia et al., 2018). Hence, our hypothesis was that we would observe minor structural differences as captured by voxel‐based morphometry but more extensive regions of textural differences between APOE4 carriers and non‐carriers, which could potentially constitute a prelude for future atrophy. An additional aim of the study was to determine how age and sex (with older age and female sex related to higher risk for AD) are related to textural features and whether there is a significant interaction between APOE4 and age in predicting texture.

## 2 MATERIALS AND METHODS

### 2.1 Cohort

The ALFA study enrolled 2743 cognitively healthy volunteers aged between 45 and 76 years (Molinuevo et al., 2016). Exclusion criteria included performance not meeting prespecified cutoffs on cognitive tests (as described in more detail in Molinuevo et al., 2016) and the presence of a psychiatric diagnosis. The ALFA study was approved by the Independent Ethics Committee ‘Parc de Salut Mar’. For the PREVENT‐Dementia programme, 700 participants were recruited from five study sites: West London (*n* = 210), Edinburgh (*n* = 222), Cambridge (*n* = 100), Oxford (*n* = 68) and Dublin (*n* = 100). The main entry criteria were age between 40 and 59 years and the absence of dementia or other neurological disorders. Approval for the study has been given by the NHS Research Ethics Committee London Camberwell St‐Giles (REC reference: 12/LO/1023), which operates according to the Helsinki Declaration of 1975 (and as revised in 1983), by the Trinity College Dublin School of Psychology Research Ethics Committee (SPREC022021‐010) and the St James Hospital/Tallaght University Hospital Joint Research Ethics Committee. All participants signed written informed consent forms. Of the recruited participants, 666 from the PREVENT‐Dementia programme and 1503 from ALFA completed an MRI scanning session.

### 2.2 MRI protocol

T1‐weighted scans were used for the present project. The PREVENT‐Dementia protocol included a magnetization‐prepared rapid gradient echo (MPRAGE) acquisition (repetition time = 2.3 s, echo time = 2.98 ms, 160 slices, flip angle = 9°, voxel size = 1 mm^3^ isotropic) using the following scanners: 3T Siemens Prisma (Oxford, Edinburgh), Prisma fit (Cambridge), Verio (West London, Edinburgh) and Skyra (Dublin; Edinburgh). The ALFA protocol included a T1‐weighted Turbo Field Echo (TFE) sequence (repetition time = 9.9 ms, echo time = 4.6 ms, flip angle = 8°, voxel size = 0.75 mm^3^ isotropic), which was acquired in a 3 T Philips scanner (Ingenia CX, Philips, Amsterdam, Netherlands). Data were visually inspected, and images were excluded due to incidental findings, poor quality or artefacts that could influence the incipient textural comparisons.

### 2.3 Textural maps

**FIGURE 1:**
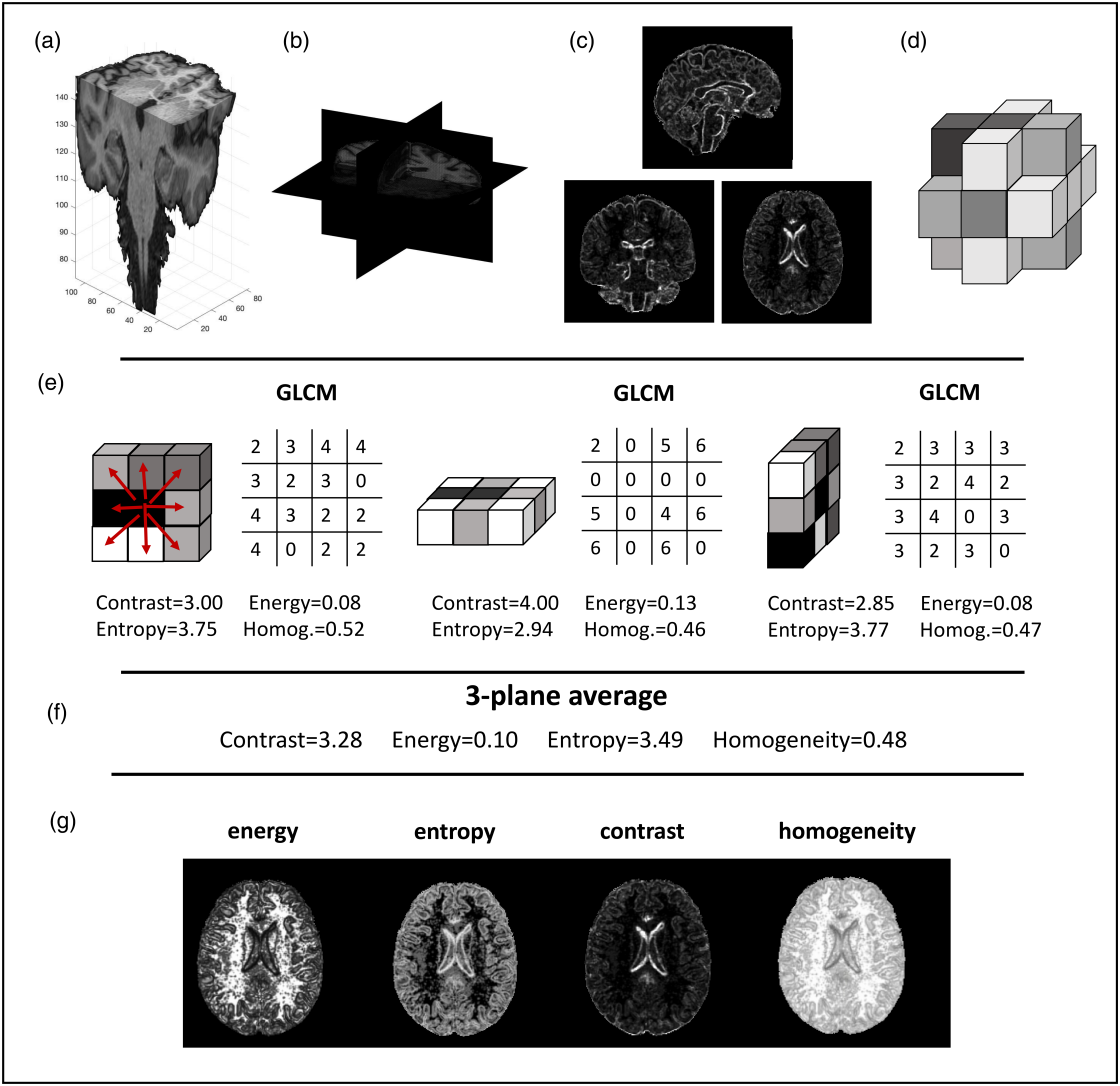
Textural map generation in three orthogonal planes. (a) A brain‐extracted bias field corrected scan is shown, depicting all three analysis planes. (b) Three orthogonal planes and three corresponding slices in these planes are shown. (c) The generated contrast maps are shown (three planes). (d) The local voxel neighbourhood on which the analysis is run involves the 18 direct neighbours of a voxel. (e) Generation of GLCM matrices and calculation of the respective textural features in three planes. (f) Calculation of the average between the three planes which ends up being the textural value of the central voxel. (g) A single axial slice for generated textural maps for the four examined textural features.

All processing was conducted in Matlab 2019a (R2019a; The MathWorks Inc., Natick, MA, USA). T1‐weighted scans were segmented onto GM, white matter (WM) and cerebrospinal fluid (CSF) using SPM12. Bias field corrected scans were saved as part of the pre‐processing. GM, WM and CSF segmentations were used to create a brain mask which was applied to the bias field corrected image. The masked images were all quantised to eight intensity levels by normalising relevant to the dynamic range of the intensity values within the masked image (graycomatrix Matlab function; the range of available image intensities was divided into equal bins). These were subsequently used to generate voxel‐wise textural maps following the published methodology (Maani et al., 2015). The analysis was run using a three‐dimensional (3D) mode and separately for the axial, coronal and sagittal planes (Figure 1). For this method, GLCMs are generated for a small voxel neighbourhood (nine voxels in this implementation) in each plane. A distance *d* = 1 between voxels, as is customary was chosen and eight directions on which the analysis was run. The GLCM method counts how many times a certain pair of the binned intensities occurs in every direction in the imaging patch where the analysis is run. A matrix (8 × 8 in the case of eight intensity levels) is constructed and subsequently, features such as contrast and homogeneity are calculated based on the published formulas for Haralick features (Haralick et al., 1973). Textural values are assigned to every voxel by calculating the features using the GLCM method within 3 × 3 voxel neighbourhoods. Subsequently, the calculated textural values for each feature are averaged between the three planes. Conceptually, features such as energy and homogeneity have higher values with a more homogeneous intensity profile, whereas features such as entropy and contrast have higher values with a more heterogeneous intensity profile. Though the features are typically highly correlated, they capture different information related to statistical relationships of neighbouring voxel intensities.

As part of the SPM12 pipeline, a study template using DARTEL was created and flow fields to register scans to and from the MNI space were generated (Ashburner, 2007). The individual textural maps were then registered to the MNI space using a 2 mm isotropic voxel and a smoothing kernel with full width at half maximum of 2 mm. The GM maps were registered to MNI space for VBM using a 4 mm kernel. The generated template and registered scans were visually assessed, and in cases where the registration was not successful, scans were excluded from further analysis.

### 2.4 Statistical analysis

Demographic variables were compared between APOE4 non‐carriers, heterozygotes and homozygotes using one‐way ANOVA for continuous variables and the *χ* ^2^ test for categorical variables.

Textural scans and GM maps in MNI space were harmonised before statistical analysis to account for site‐specific effects using voxel‐wise COMBAT harmonisation, which was applied in Matlab (Fortin et al., 2018). COMBAT was applied for all non‐zero voxels following the generation of a mask based on the registered maps. For the harmonisation, six centres were considered (one ALFA and five PREVENT sites) and age, sex, education and APOE4 carriership were included as modulators (i.e., their physiological relevance was preserved). Group comparisons were conducted with FSL's randomise (Winkler et al., 2014) to detect differences between APOE4 carriers and non‐carriers. As a further sensitivity analysis, voxel‐wise comparisons for textural features were run with unsmoothed maps registered to MNI space and with maps smoothed with a 4 mm full width at half maximum smoothing kernel. Furthermore, APOE4 was also considered as a three‐group variable (non‐carriers, heterozygotes, homozygotes) and tests investigating textural differences between two groups at a time were run. To further examine if the textural associations were the same in different sites, we repeated the textural analysis for unharmonised contrast and homogeneity data for the three bigger sites considered in this study: ALFA (956), PREVENT‐Edinburgh (204) and PREVENT‐West London (185).

The effect of age and sex on textural properties was examined with separate models, controlling for all covariates of interest (age, sex, APOE4, education years). Differential associations between age and textural properties/regional brain volume in APOE4 carriers and non‐carriers were investigated using interaction analysis.

All voxel‐wise analyses were run with 5000 permutations and were corrected for multiple comparisons using the family‐wise error rate method. Moreover, the threshold‐free cluster enhancement method was used to determine significant clusters (Smith & Nichols, 2009).

## 3 RESULTS

Data from 629 PREVENT‐Dementia participants and 956 from the ALFA study were available and of good quality. Details about the analysable cohort are shown in Table 1. Datasets were excluded due to missing demographics (11), imaging‐related artefacts and incidental findings which would impact textural analysis (553) or unsuccessful pre‐processing (20). The VBM analysis revealed no areas of volumetric difference between APOE4 carriers and non‐carriers in the cohort. Similarly, no differences were found when APOE4 carriership was examined as a three‐group variable and binary comparisons were conducted between non‐carriers, heterozygotes and homozygotes.

**TABLE 1:** Demographic specifications for the combined ALFA and PREVENT cohorts.

	APOE4 non‐carriers (964)	APOE4 heterozygotes (541)	APOE4 homozygotes (80)	*p* Value
Age (years)	56.2 ± 7.3	56.3 ± 7.7	55.0 ± 6.2	.35
Sex (% females)	63.2%	61.9%	61.3%	.86
Years of education	14.8 ± 3.8	14.8 ± 3.9	14.6 ± 3.7	.86
Cohort (% ALFA)	59.7%	61.6%	60.0%	.77

*Note*: Values shown as mean ± standard deviation and percentages.

### 3.1 Textural variations with age, sex and APOE4 genotype

**FIGURE 2:**
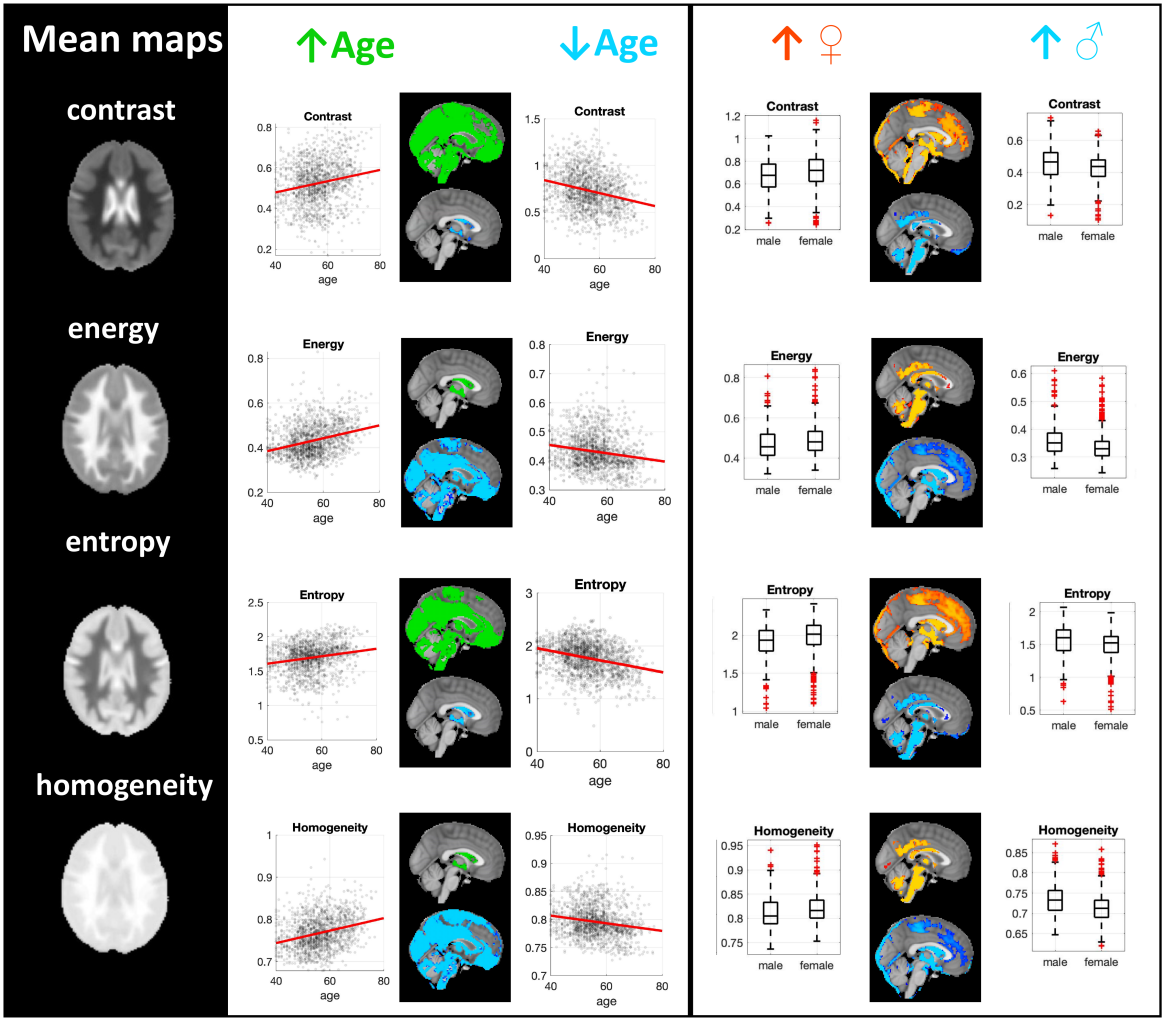
Voxel‐wise textural associations with age and sex. Mean textural maps are shown for all calculated textural features. In the second column, associations with ageing are shown. Green is used to indicate regions where ageing was associated with higher textural values, whereas blue for regions where the association was negative. Scatterplots are shown with a red line used to indicate linear fitting. In the last column, associations with sex are shown. Orange/red is used to indicate regions where females had significantly higher textural values compared to males and blue is used to indicate regions where males had higher values compared to females.

For the examined textural features, there were no significant textural differences between carriers and non‐carriers of the *APOE4* gene. There was, though, an underlying non‐significant pattern of lower homogeneity and higher contrast and entropy for APOE4 carriers (*p* = .07) for small clusters (Figure S1). There were no further underlying patterns for the conducted comparisons based on APOE4 carriership. A further exploratory analysis was undertaken to examine whether APOE4 homozygotes and heterozygotes had a different textural profile by proceeding to pairwise *t*‐tests. There were no significant clusters of difference between non‐carriers, heterozygotes and homozygotes. Ageing and female sex demonstrated distinct associations with textural features (Figure 2), especially in the following areas: for older age, higher entropy/contrast was seen in GM/WM in all lobes and higher homogeneity/energy in the ventricles and areas in the insular cortex, pre/postcentral gyrus and temporal pole. For females, sex, higher contrast/entropy was seen in the ventricles, frontal and parietal lobes, temporal pole, pre and postcentral gyri, edges of the brain and higher homogeneity/energy in WM areas, precuneus, cingulate gyrus, brainstem, cerebellum, the thalami, putamen, amygdala and temporal fusiform cortex. A more detailed depiction of the pattern of associations is shown in Figure S2.

### 3.2 APOE4 interactions with age

Further models with the interaction term ‘APOE4 × age’ were examined to detect potential areas of differential association between texture and age based on APOE4 carriership. There were no areas where the interaction between APOE4 and age was significant. Similarly, there were no areas where the interaction between APOE4 carriership and age was significant in predicting brain volume as examined with VBM.

### 3.3 Sensitivity analysis

**FIGURE 3:**
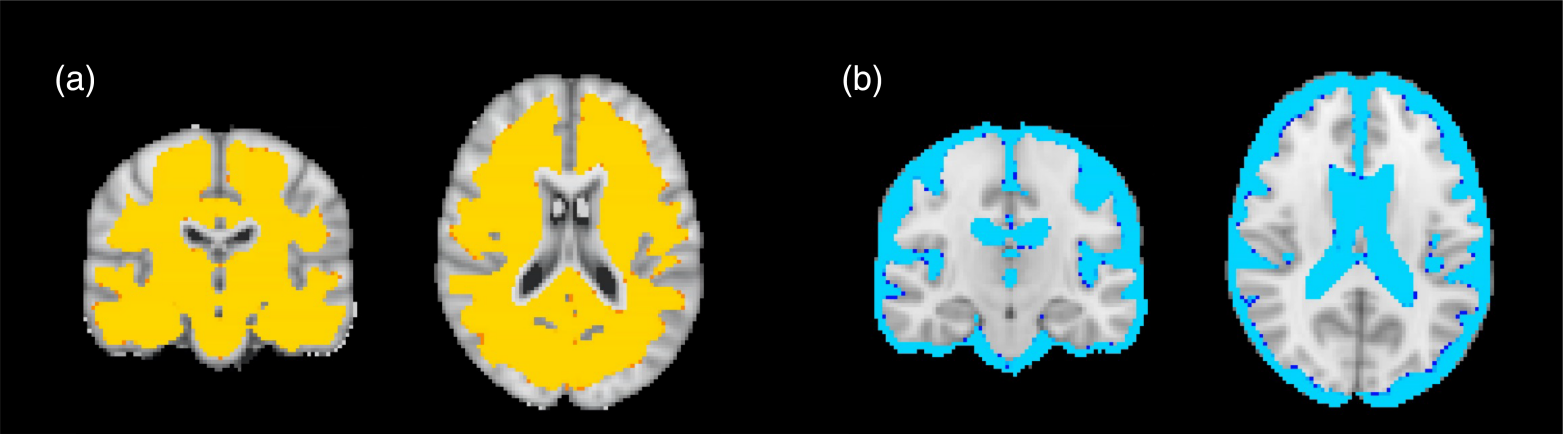
Voxel‐wise differences in texture without COMBAT harmonisation. (a) Voxels where ALFA participants had higher contrast, (b) voxels where ALFA participants had lower contrast.

To evaluate the stability of the results for the APOE4 comparisons with different smoothing kernels, the analysis was repeated with no smoothing and 4 mm smoothing. Results remained unchanged with the two alternative strategies (no areas of difference between APOE4 carriers and non‐carriers—trend towards higher entropy/contrast, lower homogeneity in a small cluster with the 4 mm kernel; similar to Figure S1). Additional analysis was run without COMBAT harmonisation to investigate the effect of the applied harmonisation approach on TBM. For the investigation of COMBAT's impact we run a voxel‐wise analysis for contrast with 2 mm smoothing using non‐harmonised data and harmonised data separately. The difference between the PREVENT and the ALFA studies was investigated. When the data were harmonised, there was no difference between the ALFA and PREVENT studies. Without harmonisation, though, extensive patterns of difference were observed between the PREVENT and ALFA datasets, where ALFA participants had higher contrast in WM and lower in GM/CSF, as in Figure 3. Finally, we have looked into APOE4 group differences and associations with age and sex within the three bigger sites separately using the unharmonised textural data. Results are presented in Figure S3 and cohort demographics are shown in Table S1 (significant differences in age, sex and education years between sites). In the PREVENT‐Edinburgh cohort there are some detected group differences between APOE4 carriers and non‐carriers in scattered regions, a finding not replicated in the whole cohort or the rest of the sites. The observed associations with age are mainly driven by the ALFA (older) cohort and the PREVENT‐West London cohort also demonstrates associations with age. For sex differences, results are again driven by ALFA, with some associations also observed for PREVENT‐Edinburgh and PREVENT‐West London.

## 4 DISCUSSION

In the present study, we evaluated textural features quantified based on typically acquired structural MRI scans in a large cognitively normal middle‐aged cohort combining the PREVENT‐Dementia and ALFA datasets. In line with our hypothesis and previous observations in the two cohorts independently, we found no prominent structural alterations in relation to APOE4 carriership when conducting voxel‐based morphometry and no significant APOE4 × age interactions when examining GM volume. Contrary to what we hypothesised, we did not find differences between APOE4 carriers and non‐carriers in textural features, and there were no significant APOE4 × age interactions in predicting texture. Textural properties were also examined in relation to ageing and sex to establish the pattern of associations. Older age was related to a more homogeneous textural profile in the ventricles and more heterogeneous in GM and WM. Female sex was connected to a more heterogeneous textural profile in the ventricles, frontal and parietal lobes and a more homogeneous profile in the brainstem, cerebellum, corpus callosum, precuneus and posterior cingulate, compared to males. We have further shown that site effects potentially affecting TBM are successfully removed with voxel‐wise COMBAT harmonisation.

VBM and TBM are two conceptually different approaches for the evaluation of brain structural alterations. In volumetry, the intensity information of every voxel is used to discern whether the voxel belongs to a certain tissue class (e.g., WM, hippocampus) and then quantify local volumes at a voxel or ROI level. In the present study, there were no volumetric differences between APOE4 carriers and non‐carriers as evaluated using VBM. Texture, on the other hand, and especially the GLCM technique, seeks to use the intensity information of every voxel to investigate co‐occurring intensity patterns. Considering that the MRI signal intensity reflects underlying tissue properties, this information can potentially capture underlying macro or microstructural alterations not captured by volumetry/morphometry. Using this approach which has shown to be more sensitive to early alterations, we did not find any evidence of textural differences between APOE4 carriers and non‐carriers or any differential associations between APOE4 and ageing. This lack of textural differences, considered in tandem with the absence of volumetric differences, suggests that structural differences (either prominent—volumetric or very subtle—textural) that could be captured with the evaluated methodologies cannot be seen at this stage and age range between APOE4 carriers and non‐carriers.

The novel 3D textural analysis method used in this project has been used in a limited number of studies to date. One study evaluated the intra‐ and inter‐site reproducibility of the technique using data from six subjects and showed that overall contrast, energy and autocorrelation demonstrated good to excellent reproducibility, especially in GM. The inter‐site intraclass correlation coefficient was lower in WM and low in the brainstem and pons (Ta et al., 2020). In the aforementioned study, there was no smoothing applied to the data. Here, we investigated differences between APOE4 carriers and non‐carriers using no smoothing and two smoothing kernels. For this particular analysis, we have shown that the effects remained null; hence absence of associations did not relate to such post‐processing choices, which further supports our conclusion of the absence of subtle structural differences at this stage between APOE4 carriers and non‐carriers. Furthermore, we have harmonised the textural maps at a voxel‐wise level; this is the first study using multi‐site textural data that has applied the COMBAT harmonisation method to TBM analysis. We have shown that COMBAT successfully removes site effects, which were very prominent when the non‐harmonised data were examined to detect site differences. In Figure 3, we showed that the ALFA cohort, which is older than the PREVENT cohort, had higher contrast in WM, which could be connected to a higher small vessel disease burden, to be expected with ageing compared to PREVENT and that the opposite pattern was observed for GM and CSF, in which areas the PREVENT cohort had higher contrast. For GM and CSF this could also be connected with differences in the extent of the areas/volumes with atrophy and ventricular enlargement to be expected with ageing. Along with ageing, differences in scanner types (Siemens vs. Philips) and coils could further lead to the amplification of between‐site differences, which are largely accounted for by the application of COMBAT harmonisation.

Group differences between APOE4 carriers and non‐carriers were not detected. There was, though, a trend towards higher contrast and entropy and lower homogeneity for small regions in the insula, and there was no dose‐dependent effect of APOE4 on texture. No previous studies have looked into textural differences in middle‐aged participants based on APOE4; however, some studies have looked into textural differences in elderly cohorts, taking into account APOE4 or amyloid and tau status. In one study looking into hundreds of textural features, eight features calculated with another textural analysis method (grey level non‐uniformity) differed between elderly MCI APOE4 carriers and non‐carriers (Zhao et al., 2020). In a study evaluating regional associations of texture with amyloid and tau PET in an elderly cohort (mean age 76 years), it has been shown that the precuneus, entorhinal, middle temporal, posterior cingulate and superior frontal cortices demonstrate higher contrast and entropy with an increasing tau burden (Lee et al., 2021). In the same study, it was shown that a higher Braak stage related to higher contrast, entropy and autocorrelation and lower cluster shade (Lee et al., 2021). Hence, these methods have demonstrated sensitivity in detecting differences in elderly cohorts with AD risk factors or pathology. The absence of such effects in the examined cohort suggests that it is potentially too early for the employed methodologies to detect such effects, which might not be present at this stage.

Our primary target was to investigate textural differences between middle‐aged APOE4 carriers and non‐carriers, given previous studies which have detected several textural differences between cognitively normal subjects and people with MCI and AD using the same 3D TBM method we used but in the ADNI cohort (Luk et al., 2018). In particular, it has been shown that controls, participants with MCI and people with AD have a different textural profile in the medial temporal lobe, hippocampi, amygdala and inferior parietal lobe. From the examined textural features in the aforementioned study, energy and homogeneity were higher in controls; they also resulted in a higher discrimination accuracy compared to other examined GLCM features when distinguishing between controls and people with AD. In the same study, it was shown that texture differed between MCI converters and non‐converters in a 3‐year time window between assessments, with the main regions of difference situated in the mesial temporal region and parietal lobes. Homogeneity was one of the textural features differing between the groups, with lower homogeneity observed for converters (Luk et al., 2018). In a study investigating the OASIS cohort, several textural differences have been found between AD and normal controls in regions such as cerebral WM, the hippocampus, thalamus and amygdala using a range of textural features, amongst them GLCM and histogram features (Chaddad et al., 2018). The hippocampus and amygdala where those regions whose textural features contributed towards higher classification accuracy. In a large study investigating a range of hippocampal textural features both cross‐sectionally and longitudinally in the ADNI cohort, it was shown that texture differed between amyloid positive and negative cognitively asymptomatic participants and that texture could predict better, future cognitive decline compared to volumetry (Wearn et al., 2023). In the study by Maani et al. introducing the method we used in the present paper (Maani et al., 2015), VGLCM‐TOP‐3D (voxel‐based GLCM with three orthogonal planes in 3D space), several GLCM features differed between AD and controls in areas such as the temporal lobe, hippocampus, anterior cingulate and corpus callosum. Contrasting these discussed studies, the present study focused on an asymptomatic middle‐aged population; we did not observe such differences based on APOE4 carriership, potentially due to the young age of the examined cohort. All the discussed studies were focused on elderly populations and populations with MCI and AD. In our exploratory analysis, though, we show that the PREVENT‐Edinburgh cohort (83/204 APOE4 carriers) demonstrated scattered areas of difference in textural features between APOE4 carriers and non‐carriers, a pattern which was not seen in the whole cohort or the other sites.

Textural features have been used in several studies investigating the classification accuracy of classifiers targeted at separating controls, MCI and people with AD or MCI‐stable from MCI‐progressors. In the CADDementia challenge spearheaded by the MICCAI community, a classifier incorporating cortical thickness, hippocampal and ventricular volume, hippocampal shape and texture achieved the highest performance (63% accuracy) in the challenge of classifying normal controls, MCI and AD (Sørensen et al., 2017). The same group has shown that hippocampal texture was better in predicting conversion from MCI to AD compared to hippocampal volume (Sørensen et al., 2016). This was true for a time window of 1 year and even more significant for a 2‐year period. In another study, textural features have been shown to predict MCI to AD conversion with higher accuracy and earlier compared to hippocampal volume (Lee et al., 2019). The ability of textural features to achieve high accuracy in predicting MCI‐progressors has been shown in further studies (Luk et al., 2018; Shu et al., 2021). In some of these classifiers, textural features have been combined with the APOE4 genotype, the inclusion of which appeared to increase the performance of the classifiers. Though textural analysis has been used in several studies examining the ability of texture to predict conversion to AD from MCI or to detect group differences cross‐sectionally, typically, hundreds of textural features are considered together. Seldom are textural features examined as stand‐alone imaging measures potentially conveying important information with clinical associations.

Though we did not find any differences in texture based on APOE4 carriership at this stage, textural features were significantly associated with age and were different between females and males for extensive regions. The observed pattern pointed towards a more heterogeneous texture in GM and WM regions with ageing and less heterogeneous in the ventricles. The female sex was associated with a more homogeneous texture in the brainstem, cerebellum, the cingulate and precuneus and the male sex with a more homogeneous profile in the ventricles and parts of the frontal and parietal lobes. The literature is scarce in terms of reports of textural associations with age and sex. A study examining anisotropy and laminarity in WM reported a more homogeneous textural profile in females and more ‘disorganised’ texture with ageing (Kovalev & Kruggel, 2007), similar to what we found in the present study in terms of both GM and WM (higher contrast and entropy, lower energy and homogeneity). Using textural descriptors, male brains have been found to be more asymmetric than female brains and increasing age was connected with both areas of higher and lower asymmetry (Kovalev et al., 2003). Texture‐based brain networks have been investigated in relation to ageing and sex. The study by da Silveira et al. (2023) investigated textural connectivity based on brain ROIs using GLCM features calculated within these ROIs (AAL) atlas. In a relatively young cohort (mean age 39 years), they found that textural connectivity differed between males and females (75/86 regions) and varied with ageing (44/86 regions). In this study (da Silveira et al., 2023) they reported that for most of the areas (amongst them the postcentral gyri, precuneus, areas in the frontal, parietal lobes and temporal pole), males tended to show higher textural similarity amongst neighbouring regions, the cerebellum was an exception; female participants showed higher similarity within it. Despite the methodological differences, this finding can be seen as in line with our findings where females show higher energy/homogeneity and lower contrast/entropy within the cerebellum.

The observed higher contrast within WM regions with ageing could potentially be explained by a higher small vessel disease burden; more specifically, white matter hyperintensities (WMH) have a darker intensity profile in a T1‐weighted image compared to the surrounding WM. In PREVENT‐Dementia, APOE4 carriers did not have a higher small vessel disease burden compared to non‐carriers (Low et al., 2022), however, in a subset of the ALFA study, the prevalence of WMH was higher in APOE4 homozygotes (Rojas et al., 2018). Another study focused purely on the lateral ventricles and investigated entropy differences between controls, MCI and AD patients using textural analysis. The study found higher entropy in MCI and AD compared to controls (Veluppal et al., 2022). In the present study, we found that there is lower entropy in areas within the ventricles with ageing and no differences when APOE4 was considered. Changes that might relate to the choroid plexus might have driven the results in the aforementioned study, where AD patients and people with MCI were examined relative to controls, as opposed to our study, where all participants were healthy. The observed higher homogeneity/lower entropy in the ventricles with ageing in our cohort was somewhat unexpected since we would have expected that ageing would relate to a more prominent choroid plexus in some subjects and, thus, the opposite pattern. One possible explanation is that registration imperfections might have led to this observation, as well as differences in the extent of the ventricles (a whole brain mask is used for the analysis, which incorporates CSF spaces as well). Another plausible explanation is that this difference in the textural profile of the ventricles could relate to different CSF flow patterns and the associated introduced noise, differences that can be investigated with other techniques such as phase contrast MR angiography (Sakhare et al., 2019). A study examining textural networks found an association between textural features and age for 44 of 86 examined regions (da Silveira et al., 2023). The age distribution of this study was very broad (18–90), with a mean age of 39 years; 257 of the 760 participants were aged between 46 and 75. The applied method in our study examined texture in the whole brain; hence, it might also be influenced by underlying differences in the extent of CSF‐filled spaces or intensity variations, especially in borderline regions between different tissue types; such effects could partially explain the observed associations with age and sex. Exploratory analysis in the bigger sites suggests that the observed effects are mainly driven by the biggest study site (ALFA), which is also the oldest. Reassuringly, some of the associations with age and female sex also exist for the PREVENT‐West London site, though for smaller regions.

T1‐weighted MRI sequences such as MPRAGE/SPGR used in the present study are not a method for T1 relaxation mapping; however, voxel intensities are related to tissue relaxation times. Hence, the textural properties quantified based on these images could relate to underlying microstructural properties. It needs to be noted that in a separate study examining the same cohort (ALFA and PREVENT) and, in particular, the orientation dispersion index in GM quantified based on single‐shell diffusion‐weighted imaging, it was found that there were no differences between APOE4 carriers and non‐carriers. It was only when interactions of APOE4 × age were investigated that significant clusters of difference were revealed (Mak et al., 2024). In this same study, no differences in cortical thickness were found between the groups, similar to what we reported in the present study for VBM. Brought together, these two studies suggest that at this age range, there are neither prominent nor subtle macro or microstructural alterations which can be detected with the described methodologies that have occurred in APOE4 carriers.

Strengths of this study include the large middle‐aged cognitively asymptomatic cohort, harmonisation approaches applied before final analysis and the generation of textural maps for evaluation of textural properties at a voxel‐wise level. Furthermore, a number of conceptually perceivable textural features were examined, and their association with ageing, sex and APOE4 was established. In addition to this, we present data both with and without smoothing. In our exploratory analysis with non‐harmonised data investigating if the same effects were seen in the three bigger sites, we observed that results were mainly driven by the ALFA cohort; this highlights both a strength of the study (the large cohort) and a limitation (variability of the results per site). Limitations of the study include the fact that only a specific set of textural features was evaluated and that further biomarkers which would aid with participant staging (fluid biomarkers) or would reveal the sensitivity of texture in detecting regional pathology (PET markers) are lacking at the moment for the full cohort. The observed associations did not replicate within sites, which could be explained by the differences in demographics between them and further emphasises the importance of applying harmonisation approaches when combining different datasets. Furthermore, the voxel‐based approach used could be considered a limitation since this renders the technique sensitive to registration imperfections, and results can be impacted, especially at the edges of different tissue types.

## 5 CONCLUSION

Overall, in this large middle‐aged cognitively asymptomatic population, we did not find evidence of volumetric or textural alterations in APOE4 carriers. We have further established textural associations with age and sex, showing a distinct textural profile with increasing age and female/male sex. Finally, we have shown that harmonisation at a voxel‐wise level is feasible with COMBAT and is essential in order to correct for site effects when TBM approaches are applied. In conclusion, at this time window, cognitively normal middle‐aged APOE4 carriers do not appear to demonstrate volumetric or textural alterations in their brains that would potentially hint towards underlying neurodegenerative processes impacting brain structure. Taken together with recent findings regarding absence of microstructural associations in the same cohort, these findings further highlight that in the examined age range, potentially irreversible structural damage cannot be detected either with microstructural imaging or with post‐processing approaches sensitive to early changes preceding volumetric alterations.

## CONFLICT OF INTEREST STATEMENT

The authors declare no conflict of interest.

## Supporting information


**Data S1.** Supporting Information.

## Data Availability

The data that support the findings of this study are available from the corresponding author upon reasonable request.
