# Supplementary material for: Texture‐based morphometry in relation to apolipoprotein ε4 genotype, ageing and sex in a midlife population
Source: Hum Brain Mapp. 2024 Jul 31;45(11):e26798. doi: 10.1002/hbm.26798 (PMC11289425; doi:10.1002/hbm.26798)
Supplement: Supplementary file 1 — Data S1. Supporting Information. [file HBM-45-e26798-s001.docx]

**Supplementary figures**

Supplementary Figure 1: Areas for which APOE4 carriers had different textural properties compared to non-carriers shown with red using a significance threshold set to p = 0.07 to observe underlying patterns as no areas were significant at a level of p = 0.05.

Supplementary Figure 2: Associations of textural features with age and female sex. A) In one example map, horizontal blue lines are used to indicate the position of the depicted slices, B) Associations with age – features are shown in each row in the following sequence: contrast, energy, entropy, homogeneity, C) higher/lower textural values with female/male sex. Green is used for positive associations with age (B) or to indicate higher values in females (C). Blue is used to indicate negative associations with age (B) and violet, higher values in males (C).

Supplementary figure 3: Textural differences between APOE4 carriers and non-carriers and associations with age and sex for the three major study sites, ALFA, PREVENT-Edinburgh (ED) and PREVENT-West London (WL). NS stands for non-significant and it means no clusters of difference were detected for contrast or homog(eneity). Blue is used for positive associations with age or means that values are higher for APOE4 carriers or females, green is used for the opposite (values lower with age or higher in APOE4 non-carriers, males).

**Supplementary Tables**

Supplementary Table 1: Demographic specifications and differences between the three larger sites included in the study. Values are shown as mean ± standard deviation or as percentages. Abbreviations: APOE4 = apolipoprotein ε4; ED – Edinburgh, WL – West London

|  | **ALFA** | **PREVENT-ED** | **PREVENT-WL** | **p-value** |
| --- | --- | --- | --- | --- |
| **Age (years)** | 59.5 ± 6.6 | 51.3 ± 5.6 | 51.3 ± 5.4 | **P<<0.01** |
| **Sex (% female)** | 62.7% | 57.8% | 71% | **0.03** |
| **Education (years)** | 13.5 ± 3.5 | 16.7 ± 3.2 | 15.9 ± 3.4 | **P<<0.01** |
| **APOE4 (% carriers)** | 39.9% | 40.7% | 37.3% | 0.77 |
